# Variations in the End-Use Quality of Whole Grain Flour Are Closely Related to the Metabolites in the Grains of Pigmented Wheat (*Triticum aestivum* L.)

**DOI:** 10.3390/plants14020171

**Published:** 2025-01-09

**Authors:** Bin Wang, Jie Kang, Shuaiqi Wang, Fasih Ullah Haider, Yingxin Zhong, Peng Zhang

**Affiliations:** 1 State Key Laboratory of Black Soils Conservation and Utilization, Northeast Institute of Geography and Agroecology, Chinese Academy of Sciences, Changchun 130102, China; wangbin01@iga.ac.cn (B.W.); wangshuaiqi@iga.ac.cn (S.W.); haider281@iga.ac.cn (F.U.H.); 2 National Technique Innovation Center for Regional Wheat Production, Key Laboratory of Crop Physiology, Ecology in Southern China, Ministry of Agriculture, Nanjing Agricultural University, No.1 Weigang Road, Nanjing 210095, China; 11122126@njau.stu.edu.cn (J.K.); yingxinzhong@njau.edu.cn (Y.Z.)

**Keywords:** pigmented wheat, lipids, amino acids, end-use quality

## Abstract

Whole grain flour is considered a part of a healthy diet, especially when produced with pigmented wheat (*Triticum aestivum*). However, the specific metabolic pathways and mechanisms by which these metabolites affect the end-use quality of pigmented wheat varieties still need to be better understood. This study examined the relationship between metabolite concentrations and the end-use quality of three wheat varieties: common wheat (CW, JM20), black wheat (BW, HJ1), and green wheat (GW, HZ148). The study’s findings revealed significant differences in the accumulation of metabolic substances among the various pigmented wheat varieties. Specifically, BW and GW exhibited notably higher levels of amino acids, derivatives, and lipids than CW. The study’s findings revealed significant differences in the accumulation of metabolic substances among the various pigmented wheat varieties. Specifically, BW and GW exhibited notably higher levels of amino acids and their derivatives and lipids than CW. Amino acid derivatives, such as glutathione and creatine, are compounds formed through chemical modifications of amino acids and play crucial roles in antioxidative defense and energy metabolism. The gliadin and glutenin content of BW increased by 12% and 2%, respectively, compared to CW, due to elevated levels of amino acids and their derivatives, whereas GW was notable for its higher globulin content (an increase of 11.6%). BW was also distinguished by its exceptionally high anthocyanin content, including cyanidin-3-O-(6-O-malonyl-beta-D-glucoside) (23.2 μg g^−1^), cyanidin-3-O-glucoside (6.5 μg g^−1^), and peonidin-3-O-glucoside (2.3 μg g^−1^), which surpassed the levels found in both CW and GW (which approached zero). However, BW had lower gluten content, resulting in a greater weakening and reduced development and stability times. Conversely, GW exhibited an increased lipid metabolism, which was associated with a higher starch and gluten content, improving the maximum tensile resistance. Overall, the pigmented wheat varieties offer superior nutritional profiles and processing advantages, necessitating further research to optimize their commercial use.

## 1. Introduction

Wheat (*Triticum aestivum*) is a globally cultivated staple, forming the basis of various food products such as biscuits, pastries, noodles, pasta, steamed bread, and baked goods [1]. As a primary source of dietary calories and protein, wheat has experienced increased yields in recent decades, owing to advancements in genetic research and agricultural technology. However, the quality of winter wheat grains, a crucial aspect of wheat production, has yet to see the same improvement. Stagnation or decline has been observed in recent years, posing a significant challenge to the global wheat industry [2,3]. Extensive ongoing research is focused on developing new or original wheat cultivars with enhanced quality and productivity. This effort emphasizes optimizing photosynthesis potential, metabolic composition, and adaptive responses to various environmental stress factors. These advancements aim to address the wheat industry’s persistent challenges while meeting global food security demands [4,5]. In addition to its essential nutrients, including proteins, carbohydrates, and vitamins, wheat contains beneficial compounds such as amino acids, lipids, and anthocyanins. Although wheat is not typically characterized by its lipid content, its unsaturated fatty acids are crucial for human health, contributing to immune function and delaying aging [6]. Simultaneously, lipids are critical in distinguishing wheat varieties and are closely linked to flour products’ quality and subsequent processing [6]. Free fatty acids can interact with amylose, influencing dough properties and potentially extending the shelf life [7].

The composition of wheat grain protein, which includes albumin, globulin, glutenin, and gliadin, is crucial for determining wheat quality [8]. Gliadin enhances dough extensibility and viscosity, while glutenin contributes to dough elasticity [8]. The protein content of the grain is closely associated with amino acid composition. For instance, specific amino acids like proline, glutamic acid, and cysteine in gliadin, and aspartic acid, arginine, and histidine in albumin significantly influence wheat quality [9]. Protein content serves as a key indicator of flour product quality. The bread volume positively correlates with the globulin, glycine, and gluten levels. In contrast, the structural characteristics of noodles—such as hardness, elasticity, and chewability—show a positive correlation with glutenin content and a negative correlation with gliadin content [10]. Moreover, protein content affects water absorption positively, whereas wet gluten content negatively impacts dough stability and flour mass [11].

Gluten, a protein component of wheat, is pivotal in assessing quality, whereas wet gluten is a marker for dough characteristics and processing attributes. Differences in gluten content affect the properties of flour-based products such as bread, noodles, and biscuits. Higher levels of gluten contribute to elastic and robust dough, while lower levels result in a more fragile and crumbly texture [11]. The gluten index, influenced by wheat varieties, quantitatively measures gluten quantity and quality [12]. The viscoelastic properties of wheat dough, influenced by the protein content of the grain, significantly impact the quality of cookies [11,13].

Wheat starch constitutes 65–75% of its dry weight and is the primary carbohydrate component, consisting of amylose and amylopectin [14]. The ratio and structure of these components are pivotal in determining food products’ texture and nutritional characteristics [14]. Amylose content explicitly impacts steamed bread’s quality, where lower levels are associated with an increased elasticity and a reduced hardness and chewiness [15]. Starch content and protein–lipid interactions are critical in shaping cereal grains’ culinary and dietary attributes. Interactions between proteins and starch during cooking can alter the rheological properties of foods, while lipids can form complexes with starch, influencing both digestibility and texture [16,17]. Pigmented wheat, available in shades such as black, purple, and green, distinguishes itself from traditional yellow or white varieties due to its distinctive nutritional composition and the associated health advantages [18]. Flour derived from pigmented wheat exhibits notably elevated levels of anthocyanins and an elevated antioxidant capacity compared to white flour. Key contributors to this enriched profile include anthocyanin-3-O-galactoside, anthocyanin-3-O-glucoside, and various other anthocyanins [19]. In addition to their nutritional benefits for humans, anthocyanins positively affect crop resistance to abiotic stress. For example, the genotypes of colored wheat with a high anthocyanin content can maintain significantly higher dry matter yields after a salt stress treatment [20].

Metabolomics, a robust method for identifying metabolic pathways and distinct metabolites in crops, has gained extensive use [7,21]. However, knowledge about the specific metabolic substances in pigmented wheat and their effects on flour quality under diverse environmental conditions still needs improvement. Therefore, this study aims to address this gap by utilizing non-targeted metabolomics to analyze the variations in metabolites, proteins, and starch between black wheat (BW), green wheat (GW), and common wheat (CW) grains. The primary objectives were to understand the impact of these metabolic differences on flour extensibility, processing characteristics, and overall quality. This research also seeks to inform the breeding strategies that enhance environmental adaptability and end-use quality for pigmented wheat varieties. It was hypothesized that the specific metabolic profiles in pigmented wheat varieties significantly influence their flour quality when compared to common wheat. The findings of this study are of utmost importance as they provide a deeper understanding of the metabolic differences in pigmented wheat while offering practical insights for improving flour product quality and fostering sustainable agricultural practices.

## 2. Results

### 2.1. Nutrient Composition

#### 2.1.1. Metabolic Profiling

**Figure 1:**
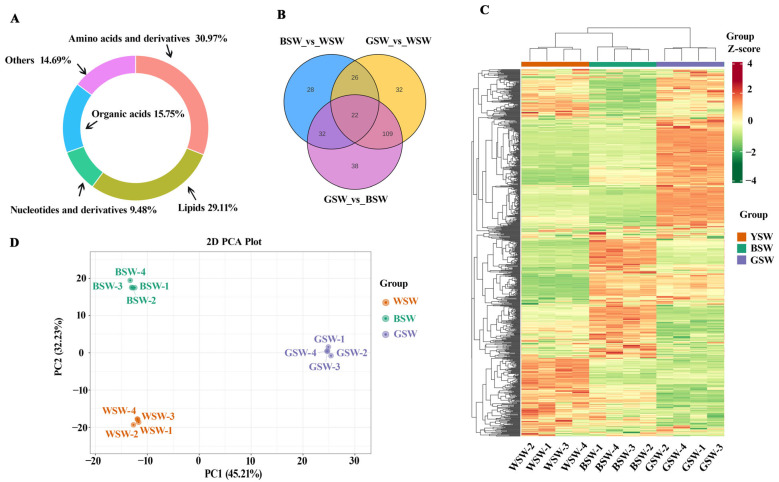
Concentrations of primary metabolites in grains of different wheat cultivars based on UPLC-MS/MS approach. (**A**) The circular graph of the metabolite classes. Each color represents a metabolite class, and the color block’s area indicates the class’s proportion. (**B**) The differential relationship between different varieties. Each circle in the figure represents a comparison group, and the numbers in the overlapping part of the circle represent the number of different metabolites shared between the comparison groups, while the numbers in the non-overlapping part represent the number of different metabolites unique to the comparison group. (**C**) Cluster analysis of all metabolite content, showing class and landscape (by metabolite). (**D**) Principal component analysis (PCA) score plots for three samples of common wheat (CW), black wheat (BW), and green wheat (GW). Four replicates per variety.

To investigate the differences in the primary metabolites of various wheat seeds, including CW, BW, and GW, ultra-high-performance liquid chromatography-tandem mass spectrometry (UPLC-MS/MS) was employed. This analysis identified 749 distinct metabolites across five classes: amino acids and their derivatives, lipids, organic acids, nucleotides and their derivatives, and others (Figure 1A). The cluster analysis demonstrated that the CW, BW, and GW metabolite profiles exhibited distinct groupings (Figure 1C). The Principal Component Analysis (PCA) further revealed a clear segregation of the CW, BW, and GW samples into clusters based on the first two principal components. The orderly arrangement of the samples in the PCA plot (Figure 1D) indicates notable metabolic differences among the wheat types.

#### 2.1.2. Differential Metabolite

Distinct patterns of metabolite expression were identified across the different wheat cultivars, revealing significant variations. The comparative analysis indicated that CW, BW, and GW had specific metabolites upregulated, with 28, 32, and 38 metabolites, respectively, as illustrated in Figure 1B. Notably, BW exhibited a significant increase in 74 metabolites compared to CW, characterized by a VIP score of ≥1, a *p*-value ≤ 0.05, and a Fold change of ≥2. GW showed an even greater increase, with 145 metabolites significantly elevated relative to CW. Additionally, BW uniquely accumulated 16 metabolites that were not present in CW and GW, 15 of which were lipid metabolites (Table S1), highlighting the critical role of fats in dietary balance, neurological function, and vitamin transport. The top 10 metabolites most accumulated in CW, BW, and GW were predominantly amino acids and lipids (Figure S1).

#### 2.1.3. Lipids

Lipids, though only a minor component in wheat flour, play a critical role in the quality of wheat-based products. They significantly impact both the baking properties and the nutritional value of these products. The current study identified that lipids, amino acids, and their derivatives were among the most accumulated metabolites in CW, BW, and GW. In CW, two lipids, 16-methylheptadecanoic acid and Palmitaldehyde, were notably more abundant compared to the other lipid metabolites (Figure S2A). BW demonstrated a higher accumulation of the top 10 lipid metabolites than CW, with only four lipids in this group (Figures S1 and S2B). GW’s top 10 accumulated metabolites consisted of seven lipids, including α-Linolenic Acid and γ-Linolenic Acid, which present significantly higher levels than the other lipid metabolites (Figures S1 and S2C). Notably, eight of the top ten lipid metabolites were common across all wheat types, indicating that differences in lipid profiles are more related to the accumulation levels than the types of lipid metabolites (Figure S2D).

#### 2.1.4. Amino Acids

Amino acids are essential components of wheat seeds, significantly influencing wheat’s protein composition and quality. In the current study, amino acids and their derivatives emerged prominently among the top 10 accumulated metabolites, highlighting their critical role and the necessity for further investigation. Notably, L-Lysine-Butanoic Acid exhibited significant enrichment across all the wheat varieties analyzed. Conversely, L-aspartic acid was particularly abundant in BW compared to CW and GW (Figure S3). BW exhibited the highest levels of amino acids and their derivatives, with CW’s levels falling between BW and GW.

### 2.2. Crude Protein and Its Components Content

Wheat grain protein is divided into four solubility-based fractions: albumin, globulin, glutenin, and gliadin [8]. Each fraction is distinct in influencing wheat dough’s nutritional quality and rheological properties, which are crucial for baking performance. An analysis in the current study revealed that CW has the highest crude protein content, followed by GW, with BW having the lowest (Table 1). However, no significant difference was observed between CW and GW regarding crude protein content. The concentration and composition of these protein fractions are key determinants of the nutritional profile of wheat and dough rheology, which are critical for baking quality. Among the wheat types, BW showed the highest glutenin content, though no significant differences were observed in the glutenin levels between the types. CW had a notably higher albumin content than the other varieties, while GW exhibited the highest globulin content. BW and GW both had significantly higher gliadin contents than CW. Although the albumin and gliadin contents were not significantly different between BW and GW, BW had an apparent advantage in gliadin and glutenin content, GW excelled in relation to globulin content, and CW had the highest albumin content.

### 2.3. Starch, Sedimentation Value, and Dietary Fiber

Wheat starch, consisting of amylose and amylopectin, is a key determinant of nutritional and industrial quality. Among the wheat types analyzed, CW had an amylose content of 386.47 mg g^−1^, BW had a content of 377.92 mg g^−1^, and GW had the highest content at 507.80 mg g^−1^. Correspondingly, the amylopectin contents were 64.94 mg g^−1^ for CW, 75.18 mg g^−1^ for BW, and 86.90 mg g^−1^ for GW (Table 1). Consequently, the significantly higher starch content in GW was associated with a notable increase in lipid metabolite accumulation (Figures S1 and S2). Higher starch content typically increases viscosity during cooking, affecting the final product’s mouthfeel and texture. The settlement value, an important measure for determining flour suitability for various baking applications, was significantly higher for GW (724.50 s) compared to BW (435.25 s) and CW (414.50 s) (Table 1). The dietary fiber content was notably higher in BW (41.98 g 100 g^−1^) and GW (40.41 g 100 g^−1^) compared to CW (37.99 g 100 g^−1^), with no significant difference observed between BW and GW.

### 2.4. Gluten Content and Extensibility

Wet gluten content (WGC) refers to the proportion of gluten remaining after the starch has been washed out of the flour, and it is a critical determinant of a dough’s elasticity and strength. Wet gluten, a viscoelastic substance, results from the interaction of gluten and alcohol-soluble proteins with water and is essential for assessing wheat’s cooking and baking quality. Wheat bran, recognized for its high dietary fiber and nutrient content, offers numerous health benefits, including improved blood sugar regulation, reduced blood lipids, enhanced intestinal health, obesity prevention, anti-aging effects, memory enhancement, and cancer prevention. This section also compares the quality differences between refined and whole wheat flour. The analysis indicates that GW flour dough exhibits the highest WGC, followed by BW dough (Table 1). Additionally, the dry gluten content in GW flour dough is significantly higher than that of the other varieties. However, no significant differences were observed in dry gluten content among the whole wheat flours. GW’s WGC is the highest, with common wheat falling between BW and GW. This suggests that GW dough has excellent elasticity, making it suitable for bread and other flour-based products. A dough’s tensile properties, such as elasticity and elongation, are crucial for its suitability for various food applications. Maximum tensile resistance and stretching energy indicate a dough’s resistance to deformation and ability to expand. GW flour and whole wheat dough demonstrate the highest tensile resistance, making them ideal for bread production. Conversely, BW dough, characterized by lower tensile resistance and higher extensibility, is better suited for noodle production. CW dough falls in the mid-range for these properties. Adding bran to whole wheat flour dough reduces its extensibility, reflecting a decrease in dough strength. Gluten’s physical properties—such as elasticity, toughness, plasticity, and extensibility—directly influence the dough handling characteristics, measured by indices like the Flour Quality Index (FQN) and stability time.

### 2.5. Farinograph Quality

**Figure 2:**
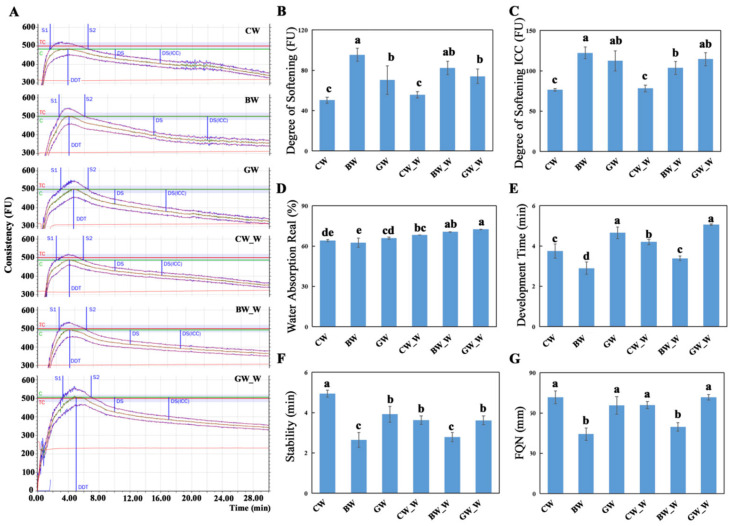
The farinograph quality of wheat flour and whole grain flour from different wheat cultivars. (**A**) Farinograph quality curve from Brabender Farinograph-TS. S1, S2—Stability; DS—Degree of Softening (10 min); DDS—Degree of Softening ICC (12 min); DDT—Development Time; CW—common wheat; BW—black wheat; and GW—green wheat. CW_W, BW_W, and GW_W indicated the wheat grain flour of different wheat cultivars. The horizontal axis is the mixing time, and the vertical axis is the dough consistency. (**B**–**G**) Farinograph of CW, BW, and GW, including (**B**) Degree of Softening; (**C**) Degree of Softening ICC; (**D**) Water Absorption Real; (**E**) Development Time; (**F**) Stability; and (**G**) FQN. The column values represent the mean ± SD of the three independent stress treatments. Different letters indicated significant differences between varieties, confirmed by the least significant difference (LSD) test (*p* < 0.05). The column values represent the means ± SD of the four independent sets of each variety.

By the GB/T 14614-2019 standard [22], the farinograph quality of refined flour and whole wheat flour from three distinct wheat varieties with different bran colors was evaluated using a Brabender farinograph from Germany. As shown in Figure 2, the water absorption rate for whole wheat flour was consistently higher across all the varieties than the rate for refined flour. While water absorption rates were relatively uniform among the refined flour doughs, the whole wheat flour dough from the GW variety demonstrated the highest absorption rate. The Farinograph Quality Number (FQN), a critical measure of a dough’s farinographic properties, was significantly higher for the refined and whole wheat flour doughs of the GW variety than for the BW variety, characterized by a lower gluten content. The GW variety exhibited the longest development times for both types of dough, reflecting its higher gluten content. In contrast, the BW variety had the shortest development times and the lowest gluten content, which aligns with the water absorption data presented in Figure 2. The common wheat variety had the highest stability times for the refined and whole wheat flour doughs. Conversely, the BW variety had significantly lower stability times, highlighting the differences in dough handling and baking properties among the varieties.

### 2.6. Anthocyanin Content

**Figure 3:**
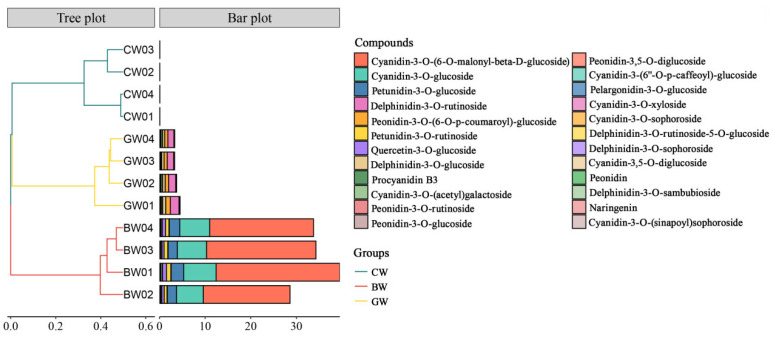
Comparative analysis of anthocyanin content among wheat types as determined by the UPLC-MS/MS method, where CW—common wheat, BW—black wheat, and GW—green wheat.

Anthocyanins are a class of water-soluble pigments widely found in plants that belong to the flavonoid compounds. In addition to giving plants bright colors, anthocyanins also have a variety of biological functions and health benefits. As depicted in Figure 3, the pigmented wheat varieties GW and BW exhibit significantly higher anthocyanin levels than CW. CW contains only peonidin-3-(6-O-p-coumaroyl)-glucoside, and its contents are very low. In contrast, BW is distinctive for the presence of naringin. Additionally, BW shows markedly higher concentrations of cyanidin-3-O-(6-O-malonyl-beta-D-glucoside), cyanidin-3-O-glucoside, and peonidin-3-O-glucoside than the other wheat varieties. GW is unique in that it contains delphinidin-3-O-glucoside and delphinidin-3-O-sambubioside. Both BW and GW share the presence of cyanidin-3-O-glucoside and procyanid in B3.

### 2.7. Correlation Analysis

**Figure 4:**
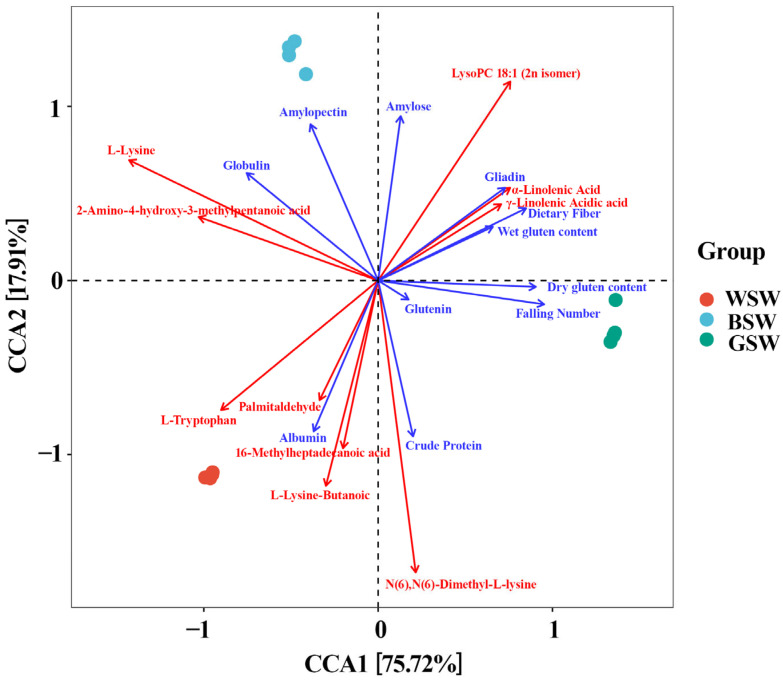
Biplot of canonical correlation analysis of nutrition and processing quality of pigmented wheat. CW—common wheat, BW—black wheat, and GW—green wheat. Four replicates per variety.

A Canonical Correspondence Analysis (CCA) was used to examine how different metabolic substances influence the quality indices of the pigmented wheat varieties. We used the CCA aims to maximize the correlation between two sets of variables (metabolites and quality parameters) by finding their respective linear combinations, thereby reflecting the overall correlation between the two sets of indicators. We selected amino acids and lipid metabolites and quality-related parameters for the correlation tests. The biplot generated from this analysis, which explains 93.63% of the total variance, provides a clear visualization of the relationships between wheat varieties and their associated metabolic substances and quality indices. In the biplot (Figure 4), the arrows represent distinct environmental factors, with their lengths indicating the magnitude of their impact. The angles between these arrows reveal the nature of their relationships: acute angles denote positive correlations, while obtuse angles indicate negative correlations. The current study analysis uncovered significant varietal differences. It was observed that with BW there is strong association between the metabolism of amino acids and their derivatives and the quality indices of globulin and starch. For GW, a significant association was found between lipid metabolism and quality indices such as gluten and the falling number. Additionally, some amino acids, derivatives, and lipids, such as albumin, were also strongly correlated with the quality index in CW. These findings were aligned with the previously observed metabolite accumulation patterns (Figure S1). For instance, the BW variety showed the highest levels of amino acids and their derivatives, as well as gliadin and glutenin, as depicted in Figure S3. Similarly, the GW variety had seven of the top ten most accumulated metabolites classified as lipids, as detailed in Figure S1.

## 3. Discussion

It is well established that the biochemical reactions of target metabolites are influenced not only by environmental factors but also by genetic factors (i.e., variety) [7]. Recently, the trend of using wheat grains of different colors has been increasing, and consumers’ attention has turned to colored wheats because they are rich in anthocyanins (natural pigments) and antioxidants. However, knowledge about the specific metabolic substances in pigmented wheats and their effects on flour quality still needs to be improved. There were significant differences in the metabolisms among the wheat varieties (Figure 1), and these results were aligned with the previous research [23], which successfully differentiated the metabolic profiles of pigmented wheat cultivars from common wheat. The observed metabolic differences can be attributed to several underlying mechanisms. For instance, variations in genetic expression among the cultivars may lead to a differential synthesis of key metabolites, such as anthocyanins in BW and GW (Figure 3), contributing to their unique health benefits and processing characteristics. The composition of the metabolites in seeds significantly influences their physiological, agronomic, nutritional, and industrial properties [24]. Metabolomic techniques, as highlighted in the literature [7,21], are essential for elucidating metabolic pathways and identifying differential metabolites across various crops. Understanding these metabolic differences not only aids in selecting wheat varieties with superior qualities but also informs the breeding strategies aimed at enhancing wheat flour’s nutritional and functional properties for various applications in the food industry.

Comparative metabolomic analyses between pigmented and common wheat varieties have highlighted significant metabolite differences, which may impact the nutritional and processing quality [23]. Previous research has shown that the pigments in soybean (*Glycine max*) seed coats can influence the isoflavone content and fatty acid composition [25,26,27]. Lipids are minor components of wheat flour and are critical to the end-use quality of wheat. The content of lipids in wheat flour is between 1.5% and 2.0%, most of which comes from the endosperm, and the rest comes from the germ and aleurone layer in tissue fragments and the oil adhering to the surface of flour grains [28]. Lipids have been shown to affect baking quality, with studies highlighting their importance for bread volume, water absorption, and gelatinization peak times [6]. BW uniquely accumulated 16 metabolites that were not present in CW and GW, 15 of which were lipid metabolites (Table S1), and GW’s top 10 accumulated metabolites consisted of 7 lipids (Figure S1). As such, the distinct lipid profiles observed in BW and GW may enhance their flour quality by improving dough elasticity and extensibility, ultimately affecting baking performance.

The elevated amylose content significantly impacts the properties of wheat flour, influencing its texture and functionality [29]. The lipids in wheat flour can be categorized into three types based on their location and extraction methods: non-starch lipids, starch lipids, and starch surface lipids [28]. The interactions between the starch, proteins, and lipids within the cereal matrix are crucial [17]. The significantly higher starch content in GW (Table 1) was associated with a notable increase in lipid metabolite accumulation. Lipids can form inclusion complexes with amylose, which impacts starch granule swelling. Non-starch lipids, especially free lipids, which include both non-polar and polar types, are crucial for the end-use quality of wheat flour [17]. This comparative analysis of lipid metabolites emphasizes their essential role in determining wheat’s nutritional and baking qualities. Understanding the accumulation patterns of the lipids in different wheat varieties can guide breeding programs to improve wheat’s end-use properties, providing practical insights for researchers and professionals in the food science, nutrition, and agricultural fields.

Additionally, protein–starch mixtures influence gelatinization behavior and water absorption [16]. Proteins affect starch gelatinization by altering thermal reactions and gel network formation, affecting the texture of cereal-based foods [30]. The nutritional quality of wheat largely depends on its protein content and amino acid profile, which are essential for its overall nutritional value [31]. BW exhibited the highest levels of amino acids and their derivatives, with CW’s levels falling between BW and GW (Figure S3). BW and GW both had significantly higher gliadin contents than CW. Although the albumin and gliadin contents were not significantly different between BW and GW, BW had an apparent advantage in gliadin and glutenin content (Table 1). These results suggest that amino acids and their derivative metabolites contribute significantly to the composition and content of proteins.

Wheat grain protein is divided into four solubility-based fractions: albumin, globulin, glutenin, and gliadin. Gliadin contributes to dough extensibility and viscosity, whereas glutenin enhances dough elasticity [8]. Wheat gluten protein is a heterogeneous mixture of an alcohol-soluble protein with a molecular weight of 30–80 kDa and polymerized glutenin with a molecular weight of 80 kDa to several million Daltons, the largest molecular weight protein found in nature. Melolin accounts for about 40–50% of the total gluten protein, and glutenin accounts for about 30–40% [28]. The specific amino acids present in each protein fraction further influence the wheat quality. For instance, proline, glutamic acid, and cysteine are prominent in gliadin; aspartic acid, arginine, and histidine are abundant in albumin; and alanine, aspartate, glycine, and cysteine are found in globulin [32]. The differences in protein composition among the wheat types are likely tied to variations in these amino acids and their derivatives. BW’s higher levels of globulin, gliadin, and amino acid derivatives (Table 1, Figure S3), underscore the importance of these factors in determining wheat quality. Additionally, non-essential amino acids such as valine, lysine, and tryptophan contribute significantly to the overall nutritional profile. Understanding the specific enrichment and balance of amino acids in the different wheat types is crucial for enhancing wheat’s nutritional quality and achieving a more balanced amino acid profile. This insight is vital for improving the overall nutritional value of wheat and optimizing its use in various food products.

A positive correlation between anthocyanin content and grain protein levels has been identified in a study of 39 colored wheat lines, suggesting a potential link between pigmentation and nutritional attributes [33]. Anthocyanin (C15H11O+, having a molecular weight of 207.24 g mol^−1^) has strong antioxidant properties, which can help combat free radical damage, reduce inflammation, and may help prevent certain types of cancer and cardiovascular diseases. BW is distinctive for the presence of naringin (Figure 3) and is known for its broad range of bioactivities, such as its antibacterial, anti-inflammatory, antiviral, and liver-protective effects [34]. BW shows markedly higher concentrations of cyanidin-3-O-glucoside and peonidin-3-O-glucoside than the other wheat varieties (Figure 3). These anthocyanins, particularly cyanidin-3-O-glucoside, are recognized for their anti-inflammatory and antioxidant properties, which contribute significantly to their potential to prevent and treat various diseases, including cancer, cardiovascular conditions, digestive disorders, and neurodegenerative diseases [35]. Furthermore, peonidin-3-O-glucoside may support bone health and be a potential intervention treatment for osteoporosis and osteomalacia [36]. Both BW and GW share the presence of cyanidin-3-O-glucoside and procyanidin B3 (Figure 3). Cyanidin-3-O-glucoside has been effective in inhibiting the growth of lung cancer cells in research studies. As a prevalent anthocyanin found in fruits and vegetables, it is noted for its antioxidant and anti-inflammatory properties. Procyanidin B3 acts as an antioxidant and holds promise as a therapeutic agent for cardiovascular diseases. Anthocyanins, in general, are highly regarded for their antioxidant properties, surpassing the efficacy of vitamins C and E in neutralizing free radicals [37]. Integrating anthocyanins from colored wheat into food processing presents significant potential for addressing metabolic disorders such as obesity, diabetes, hypertension, and dyslipidemia, highlighting their considerable health benefits [38]. Therefore, the significantly upregulated accumulation of anthocyanins, especially in BW, positively correlated with the amino acid and protein levels. This was consistent with the existing research showing that black wheat (BW) has a higher protein content, more dietary fiber, more calcium, more vitamin K, a higher total flavonoids content (TFC), a higher phenolic content (TPC), and more antioxidant activity than traditional yellow wheat. The high anthocyanin content in BW, particularly cyanidin-3-O-glucoside and peonidin-3-O-glucoside, contributes significantly to its potent antioxidative properties. These compounds are highly effective in neutralizing free radicals, surpassing the efficacy of vitamins C and E, and play a critical role in reducing oxidative stress. The consumption of whole BW has been shown to prevent cardiovascular disease, inflammation, cancer, diabetes, obesity, and aging and protect the large intestine. Anthocyanins in BW also exhibit anti-inflammatory effects by modulating cytokine activity. For instance, clinical trials have demonstrated that a BW-based diet can prevent the increase in interleukin (IL)-6 levels caused by type 2 diabetes and the tumor necrosis factor (TNF)-α, which are key inflammation markers [39]. Additionally, anthocyanins have been linked to neuroprotective effects and may help mitigate the risk of neurodegenerative diseases such as Alzheimer’s. The presence of these bioactive compounds in BW highlights its potential as a functional food ingredient for addressing metabolic disorders like hypertension and dyslipidemia.

Wet gluten, a viscoelastic substance, results from the interaction of gluten and alcohol-soluble proteins with water and is essential for assessing wheat’s cooking and baking quality. The ductility of gluten refers to the ability of gluten not to break during stretching, and it is an important index of gluten quality. BW has a significant WGC and maximum tensile resistance, which are positively correlated with its lipid levels (α-Linolenic Acid and γ-Linolenic Acid) (Table 1, Figure 3). In food processing, different flour products have different requirements for gluten ductility. For example, making bread often requires flour with good elasticity and malleability to ensure the bread has the desired volume and texture.

**Figure 5:**
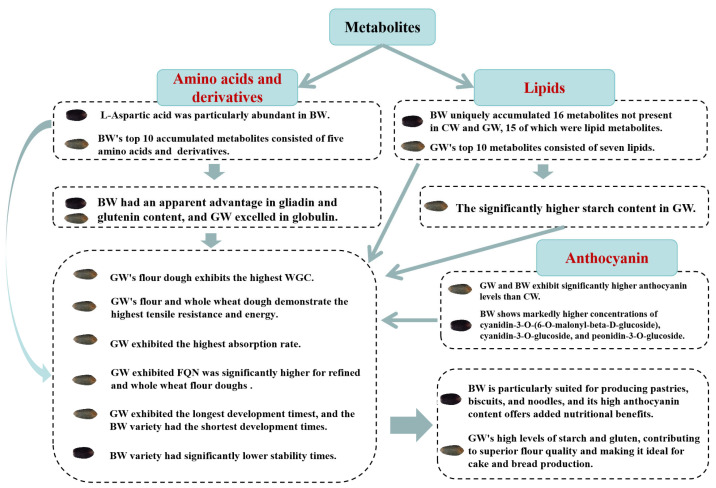
A schematic representation of the metabolites, nutritional components, and end-use quality potential indicators in pigmented wheat (*Triticum aestivum* L.). CW—common wheat, BW—black wheat, GW—green wheat, and FQN—Farinograph Quality Number.

The water absorption rate for the whole wheat flour was consistently higher across all the varieties than that of the refined flour (Figure 2). This is due to the higher dietary fiber content in the bran, which contains numerous hydrophilic groups, including hydroxyl groups [40]. A higher water absorption rate generally correlates with increased gluten formation [41]. The Farinograph Quality Number (FQN), a critical measure of a dough’s farinographic properties, was significantly higher for the refined and whole wheat flour doughs of the GW variety than those of the BW variety, characterized by a lower gluten content. This finding is consistent with the observed gluten contents. Dough development time, a key parameter that indicates the rate at which a gluten network forms in a dough, is influenced by the presence of gluten and proteins [42]. Dough stability time measures a dough’s resistance to mechanical stress during mixing, with longer times indicating a greater toughness and better processing characteristics [43]. The common wheat variety had the highest stability times for the refined and whole wheat flour doughs (Figure 5). Conversely, the BW variety had significantly lower stability times, highlighting the differences in dough handling and baking properties among the varieties. These findings have practical implications for the food industry, as they can guide the selection of wheat varieties for specific products, improving product quality and consumer satisfaction. The natural pigments in colored wheat, such as anthocyanins, can be used as food additives and natural colorants, offering aesthetic and health benefits. Colored wheat contains a variety of beneficial substances for the human body, such as protein and amino acids, and has a high nutritional value with its medicinal and health-promoting properties. In addition to its use in traditional baked goods like bread, pastries, mooncakes, and biscuits, colored wheat can be utilized in a broader range of food applications. For instance, it has the potential to develop innovative products such as functional beverages, plant-based protein supplements, and fortified snacks. Furthermore, its high anthocyanin content makes it suitable for creating natural food dyes for confectionery and dairy products. Colored wheat has already been processed into functional and nutritional products, such as selenium-rich colored wheat flour, noodles, bread, pastries, and wine, becoming a part of local specialty products. Beyond food applications, its unique nutritional composition opens opportunities for industrial uses such as the bioactive packaging of materials enriched with antioxidants or as a source of natural compounds for nutraceuticals. For example, black wheat soy sauce demonstrates its versatility in fermented foods, while its antioxidant properties could be leveraged in cosmetic or pharmaceutical formulations. These products increase the diversity of food and enhance its nutritional value and agricultural benefits. Expanding its applications to include non-food industries could further improve its market potential and contribute to sustainable farming practices.

## 4. Materials and Methods

### 4.1. Plant Materials and Growth Conditions

Three cultivars of wheat were examined to evaluate grain metabolites, flour nutritional quality, and processing characteristics: Common wheat (JM20), Black wheat (HJ1), and Green wheat (HZ148). The experiment was conducted in 2023 in Liaoheyuan Town, Jilin Province, China (42°56′ N, 124°52′ E; elevation 530.6 m above sea level). The experiment was laid out as a randomized block design with three replicates. The soil in the experimental field is classified as black soil (Chinese soil classification) with a pH of 7.1. The key soil properties include an organic carbon content of 10.98 g kg^−1^, a total nitrogen content of 1.61 g kg^−1^, an available nitrogen content of 0.141 g kg^−1^, an available phosphorus content of 0.0628 g kg^−1^, and an available potassium content of 0.147 g kg^−1^. Before planting, a basal fertilizer was applied, consisting of 210 kg of nitrogen hm^−2^, 84 kg of phosphorus hm^−2^, and 75 kg of potassium hm^−2^. Wheat sowing occurred in April 2023, and harvesting occurred in August of the same year. The harvested seeds were further used for research on wheat quality and other aspects. During this period, the average low temperature was 14–19 °C, the average high temperature was 24–29 °C, and the average precipitation was about 50 mm.

### 4.2. Determination of Metabolome Substances and Anthocyanin Content

The SYMFJ70*30 experimental milling machine from LVBO INSTRUMENT Co., Ltd., Hangzhou, China, was used to powder the colored wheat grains (with a moisture content not exceeding 15%), and the prepared flour was used for a quality analysis of factors such as HPLC and dough rheological properties. The chromatographic separation utilized an Agilent SB-C18 column (1.8 µm, 2.1 mm × 100 mm). The mobile phase consisted of Phase A, ultra-pure water with 0.1% formic acid, and Phase B, acetonitrile with 0.1% formic acid. The elution gradient was programmed: starting with 5% in Phase B at 0.00 min, increasing linearly to 95% over 9.00 min, maintaining 95% for 1 min, then returning to 5% for 10 to 11 min, followed by re-equilibration until 14 min passed. The flow rate was 0.35 mL min^−1^, and the column temperature was constant at 40 °C. Each injection volume was 2 μL. The standard and sample solutions underwent a sequential injection into the liquid chromatography-mass spectrometer (LC-MS) system. Qualitative and quantitative data analyses were conducted using the latest version of Analyst software, version 1.6.3.

### 4.3. Extraction of Crude Protein and Protein Components

The determination of crude protein content in the pigmented wheat flour was conducted with the utmost thoroughness, following the protocol outlined in the GB 5009.5-2016 standard [44]. A 0.2 g sample was placed into a 300 mL Kjeldahl flask, moistened with a few drops of water, and fortified with a Kjeldahl catalyst tablet and 5 mL of concentrated sulfuric acid for digestion. After one 1 h of digestion, the volume was adjusted to 50 mL to prepare the aliquot for analysis. A 5 mL aliquot was then pipetted and introduced into the distillation unit of the Kjeldahl nitrogen analyzer for a protein content determination. For the extraction of distinct protein fractions, albumin, globulin, prolamin, and glutelin, we utilized different extraction solvents: distilled water, a 10% sodium chloride solution, 75% ethanol, and a 0.2% sodium hydroxide solution. Each supernatant was lyophilized overnight at 130 °C to determine the residual protein content, calculated as nitrogen multiplied by a factor of 5.85. The protein yield extracted at each stage was expressed as a percentage of the protein originally present in the material, referred to as the protein yield [45].

### 4.4. Determination of Starch Content, Sedimentation Value, and Dietary Fiber

To prepare the starch from the flour, a precise amount of 100 g of flour was meticulously mixed with 60 mL of water until a smooth consistency was achieved. The dough was then carefully wrapped in plastic and allowed to rest at room temperature for 30 min. After this resting period, the dough underwent a meticulous washing process with incremental additions of 30 mL of distilled water, totaling approximately 600 mL. The resulting starch slurry was sieved through a 200-mesh sieve to separate and discard the gluten. Subsequently, the starch slurry was subjected to precise centrifugation at 4000 rpm for 15 min. Following centrifugation, the supernatant was carefully decanted, and any light-yellow substance atop the pellet was removed and discarded. The starch was then collected and subjected to filtration using a circulating water vacuum pump, with anhydrous ethanol added during filtration to wash the starch repeatedly until it attained a fluffy powder consistency. Once filtration was completed, the starch was dried in an oven set at 39 ± 1 °C for 12 h. The dried starch was finely ground with a mortar and passed through a 100-mesh sieve to obtain a sifted starch of fine consistency.

For the sample analysis, the material was first crushed through a 60-mesh sieve and degreased using ether. Approximately 0.1 g of the degreased sample was accurately weighed (to within 1 mg) and transferred into a 50 mL volumetric flask. To this, 10 mL of a 0.5 mol L^−1^ KOH solution was added, and the mixture was heated in a boiling water bath for 10 min. After cooling, the volume was adjusted to 50 mL with distilled water. Two separate 2.5 mL aliquots of this solution, one representing the sample and the other serving as a blank, were pipetted into separate containers. Each aliquot was diluted with 30 mL of distilled water, and the pH was adjusted to approximately 3.5 using a 0.1 mol L^−1^ HCl solution. An iodine reagent (0.5 mL) was added to the sample aliquot. The purpose of the iodine reagent is to react with the starch in the sample, producing a characteristic blue–black color. The blank received no iodine. Both solutions were then brought to a final volume of 50 mL and allowed to stand for 20 min for color development, which was compared against the blank.

According to the GB/T 10361-2008 standard [46], the sedimentation value of the colored wheat flour was determined using an FN-II falling number instrument (LVBO INSTRUMENT Co., Ltd., Hangzhou, China). The sedimentation value measures the flour’s quality, indicating its gluten strength and baking performance. The procedure began once the instrument was powered on and the water bath reached boiling. The flour sample was weighed based on moisture content (using 7 g of flour with 15% water content as the standard) and was placed into a viscosity tube. To this, 25 mL of distilled water at 22 ± 2 °C was added, the tube was sealed with a rubber stopper, and the contents were rapidly mixed. The viscosity tube was then positioned in the equipment to determine the sedimentation value.

### 4.5. Determination of Dietary Fiber

The wheat grains were adjusted to the appropriate moisture levels based on their moisture content, then crushed and thoroughly mixed through a 40-mesh sieve. A precise amount of the prepared flour was combined with distilled water at a ratio of 1:10 (mass to volume) in a colloid mill for 25 min to form a uniform slurry. This mixture was filtered through a 60-mesh sieve and continuously rinsed with clean water until the effluent became colorless, indicating the thorough removal of impurities. The flour was subsequently dried. The dried flour was reconstituted with distilled water at a 1:10 (mass to volume) ratio in a beaker in a constant temperature water bath set to 95 °C for 20 min to achieve a boiling state. After hydration and expansion, the mixture was cooled to 60 °C and adjusted to a pH of 5.6 using one mol L^−1^ HCl and 0.5 mol L^−1^ NaOH solutions. Heat-stable α-amylase (1.5% *w*/*w*) was then added, and the mixture was stirred at a constant temperature for 30 min to facilitate enzymatic hydrolysis. The completeness of starch degradation was confirmed using an iodine solution test. Upon reaching 50 °C, the pH of the solid–liquid mixture was adjusted to 9.0 using a two mol L^−1^ NaOH solution. Alkaline protease (3% *w*/*w*) was introduced, and the mixture was stirred at a constant temperature for 120 min. The treated mixture was thoroughly rinsed with clean water until the effluent was clear. The remaining material was dried and underwent further dry treatment. The dried flour and dietary fiber were processed using a universal crusher, passed through a 100-mesh sieve, and stored in a sealed container at room temperature in a cool, dry place. Following the GB/T 5009.88-2014 standard [47], the dietary fiber content of the pigmented wheat was determined.

### 4.6. Determination of Gluten Content

Following the GB/T 5506.2-2008 [48] and GB/T 5506.4-2008 [49] standards, the MJ-III dual-head gluten tester from LVBO INSTRUMENT Co., Ltd., Hangzhou, China, was employed to evaluate the gluten quality of both the flour and whole wheat flour obtained from the colored wheat varieties. The procedure began by setting up a fine washing screen inside a cup and ensuring it was thoroughly wetted to establish a capillary water bridge, preventing flour loss. A precisely weighed sample of 10 g ± 0.01 g was then gently transferred into the washing cup, forming an even layer of powder through gentle agitation. Using a graduated pipette, 4.8 mL of a sodium chloride buffer solution (20 g L^−1^ concentration) was carefully added uniformly and gradually to the sample. The washing cup was securely positioned on the apparatus’s bracket, and upon pressing the mixing and washing keys, the instrument commenced its automated cycle, efficiently completing the process after mixing for 20 s and washing for 5 min (for whole wheat flour, the fine screen was replaced with a coarse one during this phase).

The resulting gluten was extracted and underwent centrifugation within a sieve box. The wet gluten was weighed to determine its moisture content, and the wet gluten yield was calculated accordingly. Following the washing phase, the gluten was immediately heated in a dryer for 300 s. The dried gluten was then weighed to determine its dry weight, from which the dry gluten content was calculated.

### 4.7. Determination of Farinographical Properties

The Farinograph^®^-TS from Brabender, Duisburg, Germany, was utilized to evaluate the flour and whole wheat flour derived from the pigmented wheats, following the guidelines outlined in GB/T 14614-2019 [22]. The instrument’s water bath was activated and maintained at a controlled temperature of 30 °C throughout the analysis. A 300 g sample of flour (or whole wheat flour), standardized with a moisture content of 14%, was carefully weighed and placed into the kneading bowl of the flour analyzer. The key parameters assessed included the following: the Silty Quality Index: measures the hydration properties of the flour; the Dough Formation Time: indicates the time taken for the dough to form during kneading; the Dough Stability Time: measures the duration the dough maintains its structure under constant mixing; and the Degree of Dough Weakening: reflects the extent to which the dough loses its structure and strength over time. These parameters provide essential insights into a flour’s rheological properties and baking quality, which can directly impact the quality and consistency of your baked goods, aiding in understanding its suitability for various baking applications.

### 4.8. Determination of Extensibility

In compliance with the GB/T 14615-2019 [50] standard, the HZL-350 electronic dough extensibility tester from LVBO INSTRUMENT Co., Ltd., Hangzhou, China, was utilized to evaluate the dough characteristics of pigmented wheat flour and whole wheat flour. Initially, the Farinograph^®^-TS from Brabender, Germany, was employed to mix the flour (or whole wheat flour), water, and salt solution according to specified proportions for 5 min. Subsequently, the resulting dough was divided into two equal portions, each weighing 150 g ± 0.5 g, using scissors. These portions were shaped into spherical dough balls with a ball kneader and then removed from the device. The dough balls were rolled into strip shapes using a rolling machine and allowed to relax in a dough relaxation box. After the relaxation period, the strips were placed on the force-measuring bracket of the HZL-350 tester, and the testing procedure was initiated. The assessment continued until the sample experienced a rupture. The key indices measured during this evaluation, such as Maximum Tensile Resistance, and Extensibility, are of utmost importance as they provide valuable insights into a dough’s elasticity, strength, and overall extensibility, which are essential for assessing its suitability for various baking and food processing applications.

### 4.9. Statistical Analysis

A data analysis was performed using the single-factor Analysis of Variance (ANOVA) within the SPSS software suite (version 17.0, SPSS Inc., Chicago, IL, USA), and metabolomic and multivariate studies were performed with R (v.3.2.2; R Core Team, 2012). Dunnett’s Multiple Range Test (DMRT) was applied to further examine the distinct treatment effects and individual outcomes, with statistical significance indicated at the *p* < 0.05 threshold. Each analysis was conducted in triplicate to ensure reliability, and the results are presented as the mean ± standard deviation. The benchmark for statistical significance was set at the conventional alpha level of *p* < 0.05.

## 5. Conclusions

This study highlights the distinct effects of metabolites on the quality attributes of pigmented wheat varieties compared to conventional yellow wheat, which is commonly used in human diets. Metabolic profiling revealed that the BW variety is characterized by significantly higher concentrations of amino acids and their derivatives and an increased accumulation of lipids. These elevated amino acid levels enhance BW’s gliadin and glutenin content, though the variety has a lower gluten content and is marked by a greater weakening and reduced development and stability times. Consequently, BW is particularly suited for producing pastries, biscuits, and noodles, and its high anthocyanin content offers added nutritional benefits. In contrast, the GW variety exhibited high levels of starch and gluten, contributing to a superior flour quality and making it ideal for cake and bread production. Pigmented wheat varieties enrich the nutritional profile of wheat with additional amino acids, lipids, and anthocyanins, offering distinct advantages for various baking applications. This study also emphasized that metabolic substances, particularly amino acids, lipids, and anthocyanins, significantly impact flour quality parameters such as development and stability. However, the precise effects of these metabolites on flour quality were not fully determined in this research. Future studies should include a broader range of samples, such as more color types of wheat and more quality parameter, to provide a more comprehensive evaluation.

## Figures and Tables

**Table 1:** Nutritional composition and processing quality of pigmented wheat (*Triticum aestivum*). CW—common wheat, BW—black wheat, GW—green wheat; DW—Dry Weight; and W—Wheat variety of different whole wheat flours. Data are expressed as means ± SD (n = 8). Different superscripts a, b, c, d, and e are significantly different.

Nutritional Composition	Sample	Protein					Starch		Sedimentation Value (s)	Dietary Fiber (g 100 g^−1^ DW)
		Crude Protein (g 100 g^−1^ DW)	Albumin (mg g^−1^ DW)	Globulin (mg g^−1^ DW)	Gliadin (mg g^−1^ DW)	Glutenin (g 100 g^−1^ DW)	Amylopectin (mg g^−1^ DW)	Amylose (mg g^−1^ DW)		
	CW	12.42 ± 0.03 a	5.82 ± 0.16 a	4.23 ± 0.18 b	5.53 ± 0.18 b	22.64 ± 0.96 a	386.47 ± 18.76 b	64.94 ± 2.84 c	414.50 ± 43.95 cd	37.99 ± 1.08 b
	BW	12.35 ± 0.16 a	5.38 ± 0.09 b	3.70 ± 0.05 c	6.19 ± 0.25 a	22.91 ± 0.99 a	377.92 ± 17.18 b	75.18 ± 4.75 b	435.25 ± 45.34 c	41.98 ± 0.28 a
	GW	11.94 ± 0.15 b	5.20 ± 0.13 b	4.72 ± 0.17 a	6.04 ± 0.13 a	22.51 ± 0.14 a	507.80 ± 15.02 a	86.90 ± 3.75 a	724.50 ± 46.15 a	40.41 ± 0.81 a
Processing Quality	Sample	Wet Gluten Content (%)					Dry Gluten Content (%)		Maximum Tensile Resistance (Eu)	Extensibility (mm)
	CW	36.25 ± 1.79 ^e^					13.27 ± 0.58 ^bc^		323.67 ± 21.20 cd	123.25 ± 19.31 b
	BW	39.15 ± 1.28 ^de^					13.97 ± 0.99 ^b^		224.33 ± 17.95 e	154.50 ± 23.81 a
	GW	41.31 ± 3.29 ^cd^					17.65 ± 1.35 ^a^		883.67 ± 65.32 a	68.75 ± 3.59 c
	CW_W	43.47 ± 2.30 ^c^					12.36 ± 0.78 ^bc^		345.33 ± 21.36 c	80.75 ± 9.25 c
	BW_W	51.95 ± 2.19 ^b^					13.02 ± 0.72 ^bc^		272.67 ± 22.37 de	111.00 ± 6.78 b
	GW_W	56.92 ± 3.10 ^a^					11.83 ± 1.35 ^c^		647.00 ± 7.55 b	66.00 ± 21.68 c

## Data Availability

The data that support the findings of this study are available from the corresponding authors, upon reasonable request.

## Supplementary Materials

The following supporting information can be downloaded at https://www.mdpi.com/article/10.3390/plants14020171/s1, Figure S1: Distribution of the top 10 accumulated metabolites in pigmented wheat; Figure S2: Comparative lipid profile among wheat types; Figure S3: Distribution of amino acids and their derivatives across wheat varieties; and Table S1: Upregulated differential accumulation of metabolites.
