# Supplementary material for: Variations in the End-Use Quality of Whole Grain Flour Are Closely Related to the Metabolites in the Grains of Pigmented Wheat (Triticum aestivum L.)
Source: Plants (Basel). 2025 Jan 9;14(2):171. doi: 10.3390/plants14020171 (PMC11769550; doi:10.3390/plants14020171)
Supplement: Supplementary file 1 [file plants-14-00171-s001.zip › plants-3380998-supplementary.pdf]

Fig. S1 Distribution of the top 10 accumulated metabolites in pigmented wheat, including (A) CW; (B) BW; (C) GW. CW- common wheat, BW- black wheat, GW- green wheat. The red columns represent amino acids and derivatives, the green columns represent lipids, and the blue columns represent organic acids. The column values represent the means ± SD of the four independent sets of each variety.

Fig. S2. Comparative lipid profile among wheat types. (A-C) The top 10 accumulated lipid metabolites are highlighted for each wheat type. (D) The differential relationships among the top 10 lipid metabolites show their distribution and variation between the wheat types. Identified lipids include:

#A:

1-(2,3-Dihydroxypropoxy)-3-(((2-(Dimethylamino)ethoxy)(Hydroxy)Phosphoryl)Oxy)Propan-2-yl (11Z,14Z)-Octadeca-11,14-Dienoate;

#B: 2-(2,3-Dihydroxypropoxy)-3-(((2-(Dimethylamino)ethoxy)(Hydroxy)Phosphoryl)Oxy)Propyl (11Z,14Z)-Octadeca-11,14-Dienoate;

#C:

1-(2,3-Dihydroxypropoxy)-3-(((2-(Dimethylamino)ethoxy)(Hydroxy)Phosphoryl)Oxy)Propan-2-yl Palmitate. CW- common wheat, BW- black wheat, and GW- green wheat. The column values represent the means ± SD of the four independent sets of each variety.

Fig. S3. Distribution of amino acids and their derivatives across wheat varieties. (A-C) The figure segments highlight the top 10 accumulated metabolites of amino acids and their derivatives specific to each wheat type. (D) This part of the figure illustrates the differential relationships between the top 10 amino acids and derivative metabolites, revealing their distinctive accumulation patterns among the wheat varieties. CW- common wheat, BW- black wheat, and GW- green wheat. The column values represent the means ± SD of the four independent sets of each variety.

Fig. S1

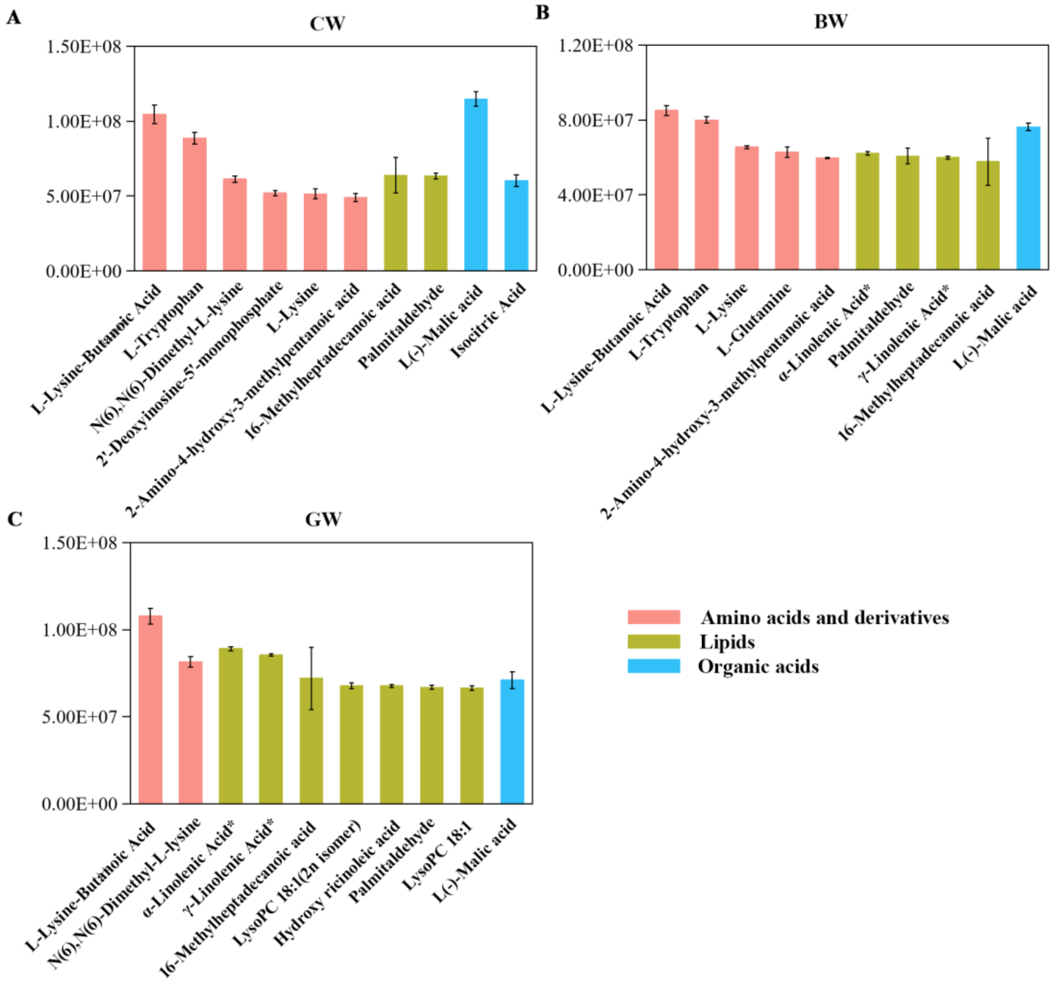
A
CW
1.50E+08
1.00E+08
5.00E+07
0.00E+00
L-Lysine-Butanoic Acid
L-Tryptophan
N(6),N(6)-Dimethyl-L-lysine
2'-Deoxyinosine-5'-monophosphate
L-Lysine
2-Amino-4-hydroxy-3-methylpentanoic acid
16-Methylheptadecanoic acid
Palmitaldehyde
L(-)-Malic acid
Isocitric Acid
B
BW
1.20E+08
8.00E+07
4.00E+07
0.00E+00
L-Lysine-Butanoic Acid
L-Tryptophan
L-Lysine
L-Glutamine
2-Amino-4-hydroxy-3-methylpentanoic acid
α-Linolenic Acid*
Palmitaldehyde
γ-Linolenic Acid*
16-Methylheptadecanoic acid
L(-)-Malic acid
C
GW
1.50E+08
1.00E+08
5.00E+07
0.00E+00
L-Lysine-Butanoic Acid
N(6),N(6)-Dimethyl-L-lysine
α-Linolenic Acid*
γ-Linolenic Acid*
16-Methylheptadecanoic acid
LysoPC 18:1(2n isomer)
Hydroxy ricinoleic acid
Palmitaldehyde
LysoPC 18:1
L(-)-Malic acid
Amino acids and derivatives
Lipids
Organic acids

Fig. S2

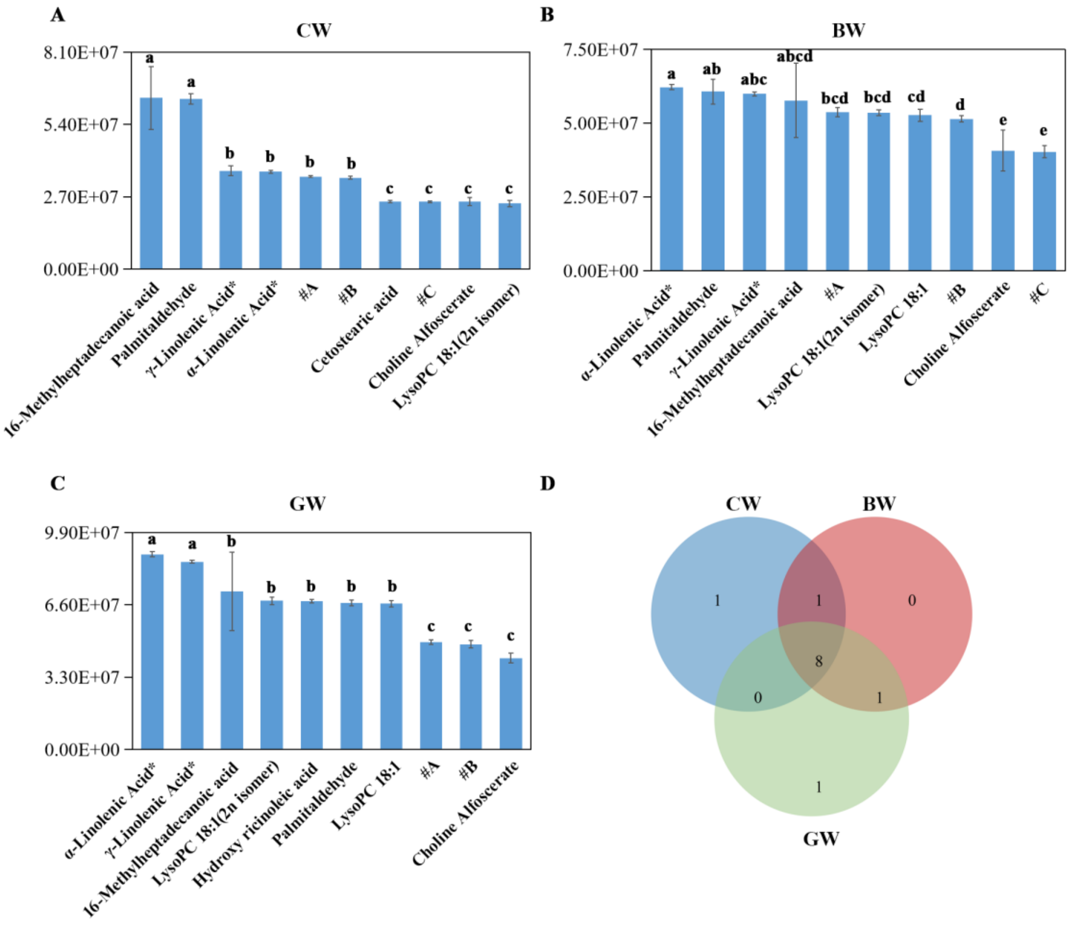
A
CW
8.10E+07
5.40E+07
2.70E+07
0.00E+00
16-Methylheptadecanoic acid
Palmitaldehyde
γ-Linolenic Acid*
α-Linolenic Acid*
#A
#B
Cetostearic acid
#C
Choline Alfoscerate
LysoPC 18:1(2n isomer)
B
BW
7.50E+07
5.00E+07
2.50E+07
0.00E+00
α-Linolenic Acid*
Palmitaldehyde
γ-Linolenic Acid*
16-Methylheptadecanoic acid
#A
LysoPC 18:1(2n isomer)
LysoPC 18:1
#B
Choline Alfoscerate
#C
C
GW
9.90E+07
6.60E+07
3.30E+07
0.00E+00
α-Linolenic Acid*
γ-Linolenic Acid*
16-Methylheptadecanoic acid
LysoPC 18:1(2n isomer)
Hydroxy ricinoleic acid
Palmitaldehyde
LysoPC 18:1
#A
#B
Choline Alfoscerate
D
CW
BW
GW
1
1
0
8
0
1
1

Fig. S3

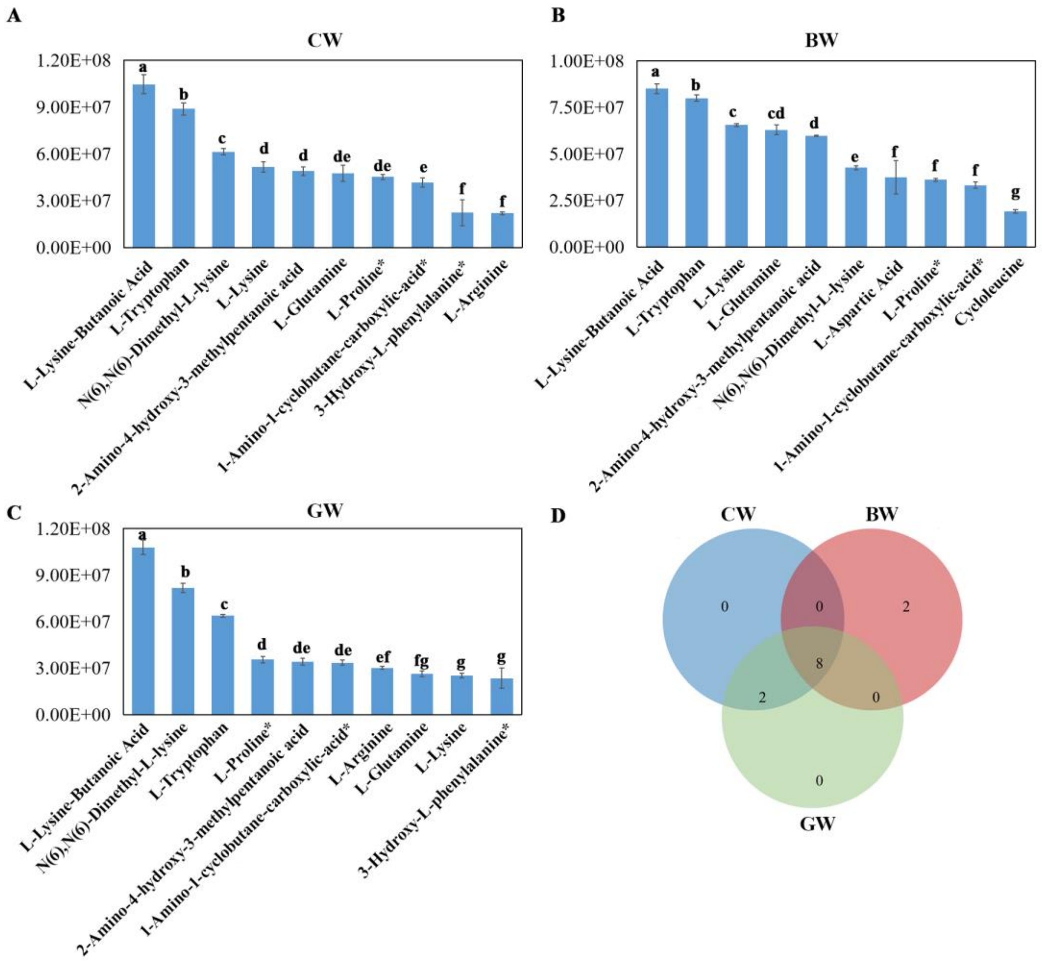
A
CW
1.20E+08
9.00E+07
6.00E+07
3.00E+07
0.00E+00
L-Lysine-Butanoic Acid
L-Tryptophan
N(6),N(6)-Dimethyl-L-lysine
L-Lysine
2-Amino-4-hydroxy-3-methylpentanoic acid
L-Glutamine
L-Proline*
1-Amino-1-cyclobutane-carboxylic-acid*
3-Hydroxy-L-phenylalanine*
L-Arginine
a b c d d de de e f f
B
BW
1.00E+08
7.50E+07
5.00E+07
2.50E+07
0.00E+00
L-Lysine-Butanoic Acid
L-Tryptophan
L-Lysine
L-Glutamine
2-Amino-4-hydroxy-3-methylpentanoic acid
N(6),N(6)-Dimethyl-L-lysine
L-Aspartic Acid
L-Proline*
1-Amino-1-cyclobutane-carboxylic-acid*
Cycloleucine
a b c cd d e f f f g
C
GW
1.20E+08
9.00E+07
6.00E+07
3.00E+07
0.00E+00
L-Lysine-Butanoic Acid
N(6),N(6)-Dimethyl-L-lysine
L-Tryptophan
L-Proline*
2-Amino-4-hydroxy-3-methylpentanoic acid
1-Amino-1-cyclobutane-carboxylic-acid*
L-Arginine
L-Glutamine
L-Lysine
3-Hydroxy-L-phenylalanine*
a b c d de de ef fg g g
D
CW
BW
GW
0 0 2 8 2 0 0

Tab. S1. Up_regulated differential accumulation of metabolites

Type	Compounds	Class I	Class II
	4-amino-5-oxo-5-(pentylamino)pentanoic acid	Amino acids and derivatives	Amino acids and derivatives
	5-Oxo-L-Proline*	Amino acids and derivatives	Amino acids and derivatives
	Ile-Phe	Amino acids and derivatives	Amino acids and derivatives
	L-Alanyl-L-Phenylalanine	Amino acids and derivatives	Amino acids and derivatives
	L-Leucyl-L-Leucine	Amino acids and derivatives	Amino acids and derivatives
	L-Serine	Amino acids and derivatives	Amino acids and derivatives
	L-Theanine	Amino acids and derivatives	Amino acids and derivatives
	L-Valyl-L-Phenylalanine	Amino acids and derivatives	Amino acids and derivatives
	Thr-Thr	Amino acids and derivatives	Amino acids and derivatives
	10,16-Dihydroxypalmitic acid	Lipids	Free fatty acids
**BW significantly upregulates accumulation compared to CW**	12-Oxo-phytodienoic acid	Lipids	Free fatty acids
	2-Dodecenedioic acid	Lipids	Free fatty acids
	3'-linoleoyl sucrose	Lipids	Free fatty acids
	5,6-DiHETrE[(±)5,6-dihydroxy-8Z,11Z,14Z-eicosatrienoic acid]	Lipids	Free fatty acids
	5S,8R-DiHODE; (5S,8R,9Z,12Z)-5,8-Dihydroxyoctadeca-9,12-dienoate	Lipids	Free fatty acids
	9,10,18-Trihydroxystearic acid	Lipids	Free fatty acids
	9,12-Octadecadien-6-Ynoic Acid	Lipids	Free fatty acids
	9,16-Dihydroxypalmitic acid	Lipids	Free fatty acids
	9-Hydroxy-12-oxo-15(Z)-octadecenoic acid*	Lipids	Free fatty acids
	9-Oxooctadeca-10,12-Dienoic Acid	Lipids	Free fatty acids
	Dodecanedioic aicd	Lipids	Free fatty acids
	Hydroperoxylinoleic acid*	Lipids	Free fatty acids
	Hydroxy ricinoleic acid	Lipids	Free fatty acids

Hydroxyicosanoic Acid	Lipids	Free fatty acids
Oleamide (9-Octadecenamide)	Lipids	Free fatty acids
Oleic acid	Lipids	Free fatty acids
octadec-2-enamide	Lipids	Free fatty acids
1-O-Linoleoyl-3-O-galactopyranosyl-L-glycerol	Lipids	Glycerol ester
1-Oleoyl-Sn-Glycerol	Lipids	Glycerol ester
1-α-Linolenoyl-glycerol-2,3-di-O-glucoside	Lipids	Glycerol ester
2-Palmitoyl-Sn-Glycerol 3-O-Diglucoside	Lipids	Glycerol ester
2-α-Linolenoyl-glycerol-1,3-di-O-glucoside	Lipids	Glycerol ester
Gingerglycolipid A	Lipids	Glycerol ester
Gingerglycolipid B	Lipids	Glycerol ester
Gingerglycolipid C	Lipids	Glycerol ester
LysoPC 18:1	Lipids	LPC
LysoPC 18:1(2n isomer)	Lipids	LPC
LysoPC 18:3	Lipids	LPC
LysoPC 18:3(2n isomer)	Lipids	LPC
LysoPC 20:1	Lipids	LPC
(3S,13Z,16Z)-3-amino-4-hydroxy-2-methyldocosa-13,16-dien-5-one	Lipids	LPE
2-(2,3-dihydroxypropoxy)-3-(((2-(dimethylamino)ethoxy)(hydroxy)phosphoryl)oxy)propan-2-yl (Z)-14-Octadecenoic Acid	Lipids	LPE
2-(2,3-dihydroxypropoxy)-3-(((2-(dimethylamino)ethoxy)(hydroxy)phosphoryl)oxy)propyl (8E,11Z,14Z)-octadeca-8,11,14-trienoate	Lipids	LPE
LysoPE 16:0	Lipids	LPE
LysoPE 16:1(2n isomer)*	Lipids	LPE
LysoPE 17:1(2n isomer)*	Lipids	LPE

LysoPE 17:1*	Lipids	LPE
LysoPE 18:0(2n isomer)	Lipids	LPE
LysoPE 18:1*	Lipids	LPE
LysoPE 18:3	Lipids	LPE
LysoPE 18:3(2n isomer)	Lipids	LPE
LysoPE 20:2	Lipids	LPE
LysoPE 20:2(2n isomer)	Lipids	LPE
LysoPE 20:5	Lipids	LPE
linolenoylethanolamine	Lipids	LPE
linoleoyl ethanolamine	Lipids	LPE
1-Arabinosyluracil	Nucleotides and derivatives	Nucleotides and derivatives
2'-O-Methyladenosine	Nucleotides and derivatives	Nucleotides and derivatives
2-(Dimethylamino)guanosine*	Nucleotides and derivatives	Nucleotides and derivatives
8-Azaguanine	Nucleotides and derivatives	Nucleotides and derivatives
Guanine	Nucleotides and derivatives	Nucleotides and derivatives
Isoguanine	Nucleotides and derivatives	Nucleotides and derivatives
N6-(2-Hydroxyethyl)adenosine*	Nucleotides and derivatives	Nucleotides and derivatives
N6-methyladenosine	Nucleotides and derivatives	Nucleotides and derivatives
Uridine	Nucleotides and derivatives	Nucleotides and derivatives
Uridine 5'-diphospho-D-glucose*	Nucleotides and derivatives	Nucleotides and derivatives
Uridine 5'-diphospho-N-acetylglucosamine	Nucleotides and derivatives	Nucleotides and derivatives
β-Pseudouridine	Nucleotides and derivatives	Nucleotides and derivatives
3-Hydroxybutyric acid	Organic acids	Organic acids
Hydroxypyruvic acid*	Organic acids	Organic acids
L-Pipecolic Acid	Organic acids	Organic acids

	Malonic acid	Organic acids	Organic acids
	D-Erythrose-4-phosphate	Others	Saccharides
	D-Pinitol*	Others	Saccharides
	4-amino-5-oxo-5-(pentylamino)pentanoic acid	Amino acids and derivatives	Amino acids and derivatives
	3'-linoleoyl sucrose	Lipids	Free fatty acids
	5,6-DiHETrE[(±)5,6-dihydroxy-8Z,11Z,14Z-eicosatrienoic acid]	Lipids	Free fatty acids
	5S,8R-DiHODE; (5S,8R,9Z,12Z)-5,8-Dihydroxyoctadeca-9,12-dienoate	Lipids	Free fatty acids
	9-Hydroxy-12-oxo-15(Z)-octadecenoic acid*	Lipids	Free fatty acids
	9-Oxooctadeca-10,12-Dienoic Acid	Lipids	Free fatty acids
	Hydroperoxylinoleic acid*	Lipids	Free fatty acids
**BW significantly upregulates accumulation compared to CW and GW**	Hydroxy ricinoleic acid	Lipids	Free fatty acids
	1-Oleoyl-Sn-Glycerol	Lipids	Glycerol ester
	1-α-Linolenoyl-glycerol-2,3-di-O-glucoside	Lipids	Glycerol ester
	2-Palmitoyl-Sn-Glycerol 3-O-Diglucoside	Lipids	Glycerol ester
	2-α-Linolenoyl-glycerol-1,3-di-O-glucoside	Lipids	Glycerol ester
	Gingerglycolipid B	Lipids	Glycerol ester
	Gingerglycolipid C	Lipids	Glycerol ester
	(3S,13Z,16Z)-3-amino-4-hydroxy-2-methyldocosa-13,16-dien-5-one	Lipids	LPE
	LysoPE 20:5	Lipids	LPE
	4-amino-5-oxo-5-(pentylamino)pentanoic acid	Amino acids and derivatives	Amino acids and derivatives
	5-Oxo-L-Proline*	Amino acids and derivatives	Amino acids and derivatives
**GW significantly upregulates accumulation compared to CW**	5-Oxoproline*	Amino acids and derivatives	Amino acids and derivatives
	Cyclo (L-Prolyl-L-tyrosine*	Amino acids and derivatives	Amino acids and derivatives
	Cyclo(D-Leu-L-Pro)*	Amino acids and derivatives	Amino acids and derivatives
	Cyclo(D-Phe-L-Pro)*	Amino acids and derivatives	Amino acids and derivatives

	Cyclo(D-Val-L-Pro)	Amino acids and derivatives	Amino acids and derivatives
	Cyclo(L-Ala-L-Pro)	Amino acids and derivatives	Amino acids and derivatives
	Cyclo(L-tyrosyl-D-proline)*	Amino acids and derivatives	Amino acids and derivatives
	Cyclo(Phe-Glu)	Amino acids and derivatives	Amino acids and derivatives
	Cyclo(Pro-Leu)*	Amino acids and derivatives	Amino acids and derivatives
	Cyclo(Pro-Phe)*	Amino acids and derivatives	Amino acids and derivatives
	Cyclo(Pro-Val)	Amino acids and derivatives	Amino acids and derivatives
	Cyclo(Ser-Pro)	Amino acids and derivatives	Amino acids and derivatives
	DL-O-tyrosine	Amino acids and derivatives	Amino acids and derivatives
	Hexanoyl-L-glycine	Amino acids and derivatives	Amino acids and derivatives
	L-Homocitrulline	Amino acids and derivatives	Amino acids and derivatives
	Lys-Leu	Amino acids and derivatives	Amino acids and derivatives
	N-(3-Indolylacetyl)-L-alanine	Amino acids and derivatives	Amino acids and derivatives
	N-Acetoxy-glucosyl-alanine	Amino acids and derivatives	Amino acids and derivatives
	N-Acetyl-L-glutamic acid	Amino acids and derivatives	Amino acids and derivatives
	N-Acetyl-L-leucine	Amino acids and derivatives	Amino acids and derivatives
	N-Acetyl-L-tyrosine	Amino acids and derivatives	Amino acids and derivatives
	N-Alpha-Acetyl-L-Asparagine	Amino acids and derivatives	Amino acids and derivatives
	N-Fructosyl Pyroglutamate	Amino acids and derivatives	Amino acids and derivatives
	N-Palmitoylglycine	Amino acids and derivatives	Amino acids and derivatives
	Pyroglutamic acid	Amino acids and derivatives	Amino acids and derivatives
	Val-Val	Amino acids and derivatives	Amino acids and derivatives
	(7Z)-Hexadecenoic acid*	Lipids	Free fatty acids
	10,16-Dihydroxypalmitic acid	Lipids	Free fatty acids
	11-Octadecanoic acid(Vaccenic acid)*	Lipids	Free fatty acids

12,13-Epoxy-9-Octadecenoic Acid	Lipids	Free fatty acids
13(S)-HODE;13(S)-Hydroxyoctadeca-9Z,11E-dienoic acid*	Lipids	Free fatty acids
13-Hydroperoxy-9Z,11E-octadecadienoic acid*	Lipids	Free fatty acids
13-Hydroxy-9,11-octadecadienoic acid*	Lipids	Free fatty acids
14,15-Dehydrocrepenynic acid	Lipids	Free fatty acids
15(R)-Hydroxylinoleic Acid*	Lipids	Free fatty acids
17-Hydroxylinolenic acid	Lipids	Free fatty acids
2-Dodecenedioic acid	Lipids	Free fatty acids
2-Hydroxy-4-methyl-3-undecanoyloxypentanoic acid methyl ester*	Lipids	Free fatty acids
2R-Hydroxyoctadecanoic Acid*	Lipids	Free fatty acids
2R-hydroxy-9Z,12Z,15Z-octadecatrienoic acid	Lipids	Free fatty acids
3'-Palmitoyl sucrose	Lipids	Free fatty acids
3'-linoleoyl sucrose	Lipids	Free fatty acids
3-Oleoyl sucrose	Lipids	Free fatty acids
5,6-DiHETrE[(±)5,6-dihydroxy-8Z,11Z,14Z-eicosatrienoic acid]	Lipids	Free fatty acids
5S,8R-DiHODE; (5S,8R,9Z,12Z)-5,8-Dihydroxyoctadeca-9,12-dienoate	Lipids	Free fatty acids
6,9,10-Trihydroxyoctadec-7-enoic acid; Sanleng acid*	Lipids	Free fatty acids
7S,8S-DiHODE; (9Z,12Z)-(7S,8S)-Dihydroxyoctadeca-9,12-dienoic acid*	Lipids	Free fatty acids
8,11-Heptadecadienoic acid	Lipids	Free fatty acids
9(10)-EpOME;(9R,10S)-(12Z)-9,10-Epoxyoctadecenoic acid	Lipids	Free fatty acids
9,10,11-Trihydroxy-12-octadecenoic acid	Lipids	Free fatty acids
9,10,13-Trihydroxy-11-Octadecenoic Acid	Lipids	Free fatty acids
9,10,18-Trihydroxystearic acid	Lipids	Free fatty acids
9,10-Dihydroxy-12,13-epoxyoctadecanoic acid	Lipids	Free fatty acids
9,12,13-TriHOME; 9(S),12(S),13(S)-Trihydroxy-10(E)-octadecenoic acid	Lipids	Free fatty acids

9,12-Octadecadien-6-Ynoic Acid	Lipids	Free fatty acids
9,16-Dihydroxypalmitic acid	Lipids	Free fatty acids
9-Hydroperoxy-9Z,11E-Octadecadienoic Acid	Lipids	Free fatty acids
9-Hydroxy-12-oxo-10(E),15(Z)-octadecadienoic acid	Lipids	Free fatty acids
9-Hydroxy-12-oxo-15(Z)-octadecenoic acid*	Lipids	Free fatty acids
9-Hydroxy-13-oxo-10-octadecenoic Acid	Lipids	Free fatty acids
9-Oxo-10E,12Z-octadecadienoic acid*	Lipids	Free fatty acids
9-Oxo-12Z-Octadecenoic acid*	Lipids	Free fatty acids
9-Oxooctadeca-10,12-Dienoic Acid	Lipids	Free fatty acids
9S-Hydroxy-10E,12Z-octadecadienoic acid*	Lipids	Free fatty acids
9S-hyroxy-10E,12E-octadecadienoic acid*	Lipids	Free fatty acids
Beta-Hydroxypalmitic Acid	Lipids	Free fatty acids
DL-2-hydroxystearic acid*	Lipids	Free fatty acids
E,E,Z-1,3,12-Nonadecatriene-5,14-diol	Lipids	Free fatty acids
Eicosadienoic acid*	Lipids	Free fatty acids
Ethyl linoleate*	Lipids	Free fatty acids
Hexadecanedioic acid	Lipids	Free fatty acids
Hydroperoxylinoleic acid*	Lipids	Free fatty acids
Hydroxy ricinoleic acid	Lipids	Free fatty acids
Methyl 12-phenyldodecanoate	Lipids	Free fatty acids
Octadec-6-enoic acid; Petroselinic acid*	Lipids	Free fatty acids
Octadeca-9,12,15-trienoic acid	Lipids	Free fatty acids
Palmitoleic Acid*	Lipids	Free fatty acids
Punicic acid (9Z,11E,13Z-octadecatrienoic acid)	Lipids	Free fatty acids
Rabdosia acid A*	Lipids	Free fatty acids

alpha-Hydroxylinoleic acid*	Lipids	Free fatty acids
α-Linolenic Acid*	Lipids	Free fatty acids
γ-Linolenic Acid*	Lipids	Free fatty acids
1-Linoleoylglycerol-2,3-di-O-glucoside*	Lipids	Glycerol ester
1-O-Caffeoyl-3-O-p-coumaroylglycerol	Lipids	Glycerol ester
1-O-Linoleoyl-3-O-galactopyranosyl-L-glycerol	Lipids	Glycerol ester
1-Oleoyl-Sn-Glycerol	Lipids	Glycerol ester
1-Palmitoyl-Sn-Glycerol 3-O-Diglucoside	Lipids	Glycerol ester
1-α-Linolenoyl-glycerol-2,3-di-O-glucoside	Lipids	Glycerol ester
1-α-Linolenoyl-glycerol-3-O-glucoside*	Lipids	Glycerol ester
2-Linoleoylglycerol-1,3-di-O-glucoside*	Lipids	Glycerol ester
2-Linoleoylglycerol-1-O-glucoside*	Lipids	Glycerol ester
2-Palmitoyl-Sn-Glycerol 3-O-Diglucoside	Lipids	Glycerol ester
2-α-Linolenoyl-glycerol-1,3-di-O-glucoside	Lipids	Glycerol ester
2-α-Linolenoyl-glycerol-1-O-glucoside*	Lipids	Glycerol ester
Gingerglycolipid A	Lipids	Glycerol ester
Gingerglycolipid B	Lipids	Glycerol ester
Gingerglycolipid C	Lipids	Glycerol ester
LysoPC 16:1(2n isomer)*	Lipids	LPC
LysoPC 16:1*	Lipids	LPC
LysoPC 18:1	Lipids	LPC
LysoPC 18:1(2n isomer)	Lipids	LPC
(3S,13Z,16Z)-3-amino-4-hydroxy-2-methyldocosa-13,16-dien-5-one	Lipids	LPE
2-(2,3-dihydroxypropoxy)-3-(((2-(dimethylamino)ethoxy)(hydroxy)phosphoryl)oxy)propan-2-yl (Z)-14-Octadecenoic Acid	Lipids	LPE

LysoPE 18:0(2n isomer)	Lipids	LPE
LysoPE 18:1*	Lipids	LPE
LysoPE 20:5	Lipids	LPE
linolenoylethanolamine	Lipids	LPE
linoleoyl ethanolamine	Lipids	LPE
Adenine	Nucleotides and derivatives	Nucleotides and derivatives
Adenosine 2'-Phosphate	Nucleotides and derivatives	Nucleotides and derivatives
Cytarabine	Nucleotides and derivatives	Nucleotides and derivatives
Cytidine	Nucleotides and derivatives	Nucleotides and derivatives
Cytosine	Nucleotides and derivatives	Nucleotides and derivatives
1-Pyrroline-4-hydroxy-2-carboxylic acid	Organic acids	Organic acids
2-Hydroxy-4-methylpentanoic acid	Organic acids	Organic acids
2-Hydroxyhexadecanoic acid	Organic acids	Organic acids
2-Hydroxyisobutyric acid*	Organic acids	Organic acids
2-Hydroxyisocaproic acid	Organic acids	Organic acids
2-Hydroxymyristic acid	Organic acids	Organic acids
2-Picolinic acid	Organic acids	Organic acids
6-Hydroxyhexanoic acid	Organic acids	Organic acids
Azelaic acid	Organic acids	Organic acids
DL-3-Phenyllactic acid	Organic acids	Organic acids
Isocitric acid-1-O-diglucoside	Organic acids	Organic acids
L-Pipecolic Acid	Organic acids	Organic acids
Muconic acid	Organic acids	Organic acids
Pyrrole-2-carboxylic acid	Organic acids	Organic acids
Sebacic acid	Organic acids	Organic acids

Suberic Acid	Organic acids	Organic acids
Tianshic acid	Organic acids	Organic acids
Tropic acid	Organic acids	Organic acids
δ-Guanidinovaleric acid	Organic acids	Organic acids
2,6-Dimethyl-7-octene-2,3,6-triol	Others	Saccharides
2-Crotonyl-3-(3-methylbutanoyl)-3'-decanoyl sucrose	Others	Saccharides
Allitol	Others	Saccharides
D-Erythrose-4-phosphate	Others	Saccharides
D-Mannitol*	Others	Saccharides
D-Sorbitol	Others	Saccharides
Dulcitol*	Others	Saccharides
Gluconic acid	Others	Saccharides
Isonicotinic acid	Others	Vitamin
Nicotinic acid (Vitamin B3)	Others	Vitamin
Riboflavin (Vitamin B2)	Others	Vitamin

Where, CW- common wheat, BW- black wheat, and GW- green wheat. Four replicates per variety.
